# Analysis of aneurysmal subarachnoid hemorrhage as a multistep process

**DOI:** 10.1111/ene.16118

**Published:** 2023-10-25

**Authors:** Ynte M. Ruigrok, Gabriel J. E. Rinkel, Han‐Sol Chang, Katharina A. M. Hackenberg, Nima Etminan, Jan H. Veldink

**Affiliations:** ^1^ Department of Neurology University Medical Center Utrecht Brain Center, University Medical Center Utrecht Utrecht The Netherlands; ^2^ Department of Neurosurgery Mannheim University Hospital, Medical Faculty Mannheim, Heidelberg University Mannheim Germany

**Keywords:** aneurysmal subarachnoid hemorrhage, genes, intracranial aneurysm aneurysm, risk factor

## Abstract

**Background and purpose:**

Aneurysmal subarachnoid hemorrhage (ASAH) is a complex disease with higher incidence in women compared to men and in Japan compared to other countries. It was hypothesized that ASAH is consistent with a multistep model of disease. The following assessments were made: (1) the number of steps needed for the disease to occur and (2) whether this number may be different in female versus male and in Japanese versus non‐Japanese patients.

**Methods:**

Incidence data were generated from a meta‐analysis on ASAH incidence until 2017, which was supplemented with a literature search from 2017 to April 2023. Age‐ and sex‐adjusted incidences per 10‐year age groups were calculated and the logarithm of age‐specific incidence against the logarithm of age was regressed with least‐squares regression.

**Results:**

In 2317 ASAH patients a linear relationship between logarithm of incidence and logarithm of age was found with a slope estimate of 3.13 (95% confidence interval 2.60–3.65), consistent with a four‐step process. Similar estimates were found for female, male, Japanese and non‐Japanese patients.

**Conclusions:**

Our results suggest that ASAH is a four‐step process, also in subgroups with higher ASAH incidence. Elucidation of the exact nature of these steps can provide important clues for identification of disease mechanisms underlying ASAH.

## INTRODUCTION

Rupture of an intracranial aneurysm results in aneurysmal subarachnoid hemorrhage (ASAH), which has a high morbidity and case fatality [1]. ASAH incidence is higher in women than in men, with 65% of patients being women [1]. There are also differences in incidence in terms of geographic regions: in Japan ASAH incidence is 28.0 per 100,000 person‐years which is much higher than in other countries in the world in which an overall incidence of 6.1 per 100,000 person‐years is observed [2]. Why women and Japanese face higher risks for ASAH is unknown.

Aneurysmal subarachnoid hemorrhage is considered a complex disease caused by a combination of modifiable clinical and genetic risk factors [3]. The most important modifiable clinical risk factors for ASAH are hypertension and smoking [1]. Recently, it was shown that many common complex diseases are consistent with a multistep model of disease, in which diseases require several distinct events, or a sequence of stages, before the disease becomes manifest [4]. Such a multistep model is a mathematical modeling approach developed by Armitage and Doll; they originally applied it in cancer epidemiology [5]. They demonstrated that if the onset of a disease requires multiple subsequent molecular steps, each step with a relatively low probability of occurring per year, then a power relationship exists between the incidence of the disease and age [5]. Here, the incidence is proportional to the product of the risks of undergoing these subsequent steps. Specifically, a logarithmic increase in incidence with age obeys a power law in which 1 less than the number of steps, *n* − 1, relates to the rate of increase in the number of steps. As a result, plotting the logarithm of age at onset and the logarithm of incidence rates will result in a straight line with slope *n* – 1 when a multistep model applies. Thus, for example, a linear relationship with an estimated slope of 4 indicates that the process leading to a disease requires an average of five subsequent molecular steps. Per disease, this number of steps may differ according to several patient characteristics, including sex [6] and country of origin [7].

It was hypothesized that ASAH is a multistep process and the number of steps needed for the disease to occur was assessed. In addition, it was assessed whether this number may be different in female versus male and in Japanese versus non‐Japanese patients.

## METHODS

The Armitage and Doll methodology [5] was applied and the logarithm of ASAH incidence against logarithm age at time of ASAH was plotted with least‐squares regression as previously described [6] using incidence rates per 100,000 person‐years in 10‐year age groups derived from a meta‐analysis on ASAH incidence until March 2017 [2]. Only studies in which incidence rates were reported by age group of 10 years, sex and country were included. The meta‐analysis [2] was supplemented with a literature search from March 2017 to 1 April 2023 using the same keywords and inclusion criteria, which yielded four additional articles [8, 9, 10, 11]. If ASAH is a multistep process the plot will be linear and will have slope of *n* − 1, that is, 1 less than the number of steps needed for disease onset. These analyses were performed for all patients together, and for female versus male and Japanese versus non‐Japanese patients. As in some cancers the logarithm of incidence and logarithm of age association is nonlinear in the older age groups [6] the analyses were also performed excluding the oldest age group (i.e., ≥85 years).

## RESULTS

**FIGURE 1:**
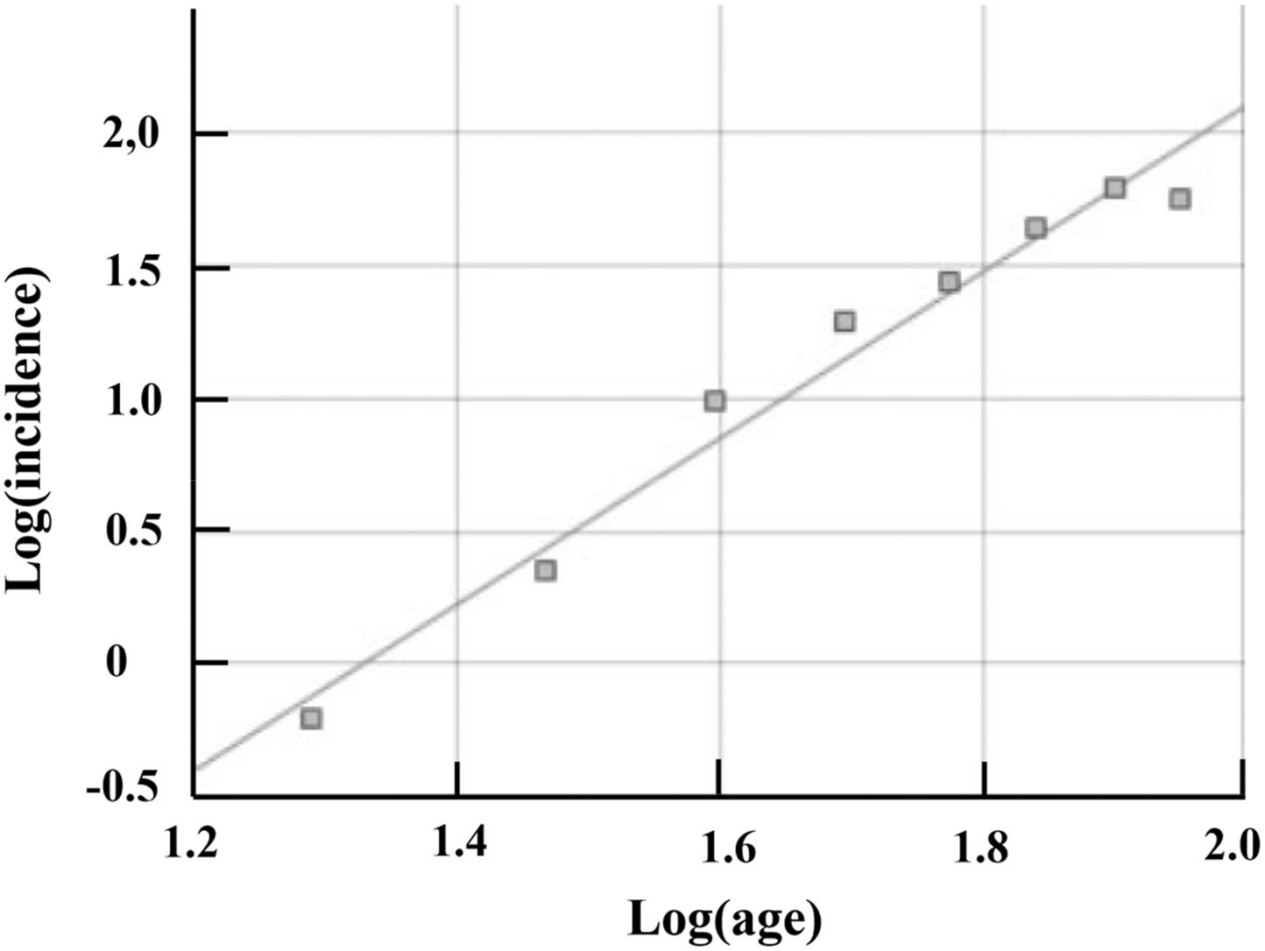
Slope estimation for all aneurysmal subarachnoid hemorrhage patients. Logarithm of incidence versus logarithm of age with slope estimate of 3.13 (95% confidence interval 2.60–3.65) and *r* ^2^ of 0.99.

On analyzing all patients (*n* = 2137) a linear relationship was found between logarithm of incidence and logarithm of age (*r* ^2^ = 0.99) with a slope estimate of 3.13 (95% confidence interval 2.60–3.65), consistent with a four‐step process (Figure 1; Table 1). Upon excluding the oldest age group the slope estimate remained essentially the same (Table 1). Similar results were also obtained when analyzing female, male and Japanese and non‐Japanese patients separately, suggesting a four‐step process in all these distinct patient groups as well (Table 1).

**TABLE 1:** Comparison of logarithm incidence versus logarithm age for different subgroups of aneurysmal subarachnoid hemorrhage patients.

	Included, *n*	*r* ^2^	Slope (95% CI)	*n* steps
All patients	2137	0.99	3.13 (2.60–3.65)	4
Excluding patients ≥85 years	1660	0.97	2.90 (2.12–3.67)	4
Female patients	1242	0.99	3.16 (2.62–3.70)	4
Male patients	895	0.97	2.93 (2.15–3.70)	4
Japanese patients	841	0.98	3.49 (2.71–4.26)	4
Non‐Japanese patients	1296	0.99	2.98 (2.48–3.47)	4

Abbreviation: CI, confidence interval.

## DISCUSSION

Our data show that the occurrence of ASAH is a process consisting of four steps. This four‐step process was also observed in the patient subgroups with a higher ASAH incidence (female and Japanese patients).

The relevant contributing steps needed for ASAH to occur can provide important clues to identify disease mechanisms but remain to be determined. In a multistep model, inheritance of genetic variations or mutations could represent a first step or first two steps, followed by the subsequent influence of modifiable clinical factors, epigenetics and potentially acquisition of somatic mutations [4, 5, 6, 7, 12]. For example, exposure to smoking, an important modifiable clinical risk factor for ASAH [1], might influence the number of steps needed to trigger a disease [4]. Smoking could enhance oxidative stress and inflammation in the arterial wall [13] but may also induce DNA methylation alterations or somatic mutations [14]. It was not possible to test the influence of smoking on the slope estimate as there were not enough ASAH incidence studies which, besides age‐, sex‐ and country‐specific data, also had data stratified by smoking. A previous study on patients with amyotrophic lateral sclerosis showed that the slope was reduced in amyotrophic lateral sclerosis patients with a mutation compared to cases without [12]. This underlines the concept of a multistep process and that mutations might have large effects [12]. Although large effect mutations for intracranial aneurysms and ASAH have been identified in family studies, these mutations do not seem to play a role on a population level [15]. Consequently, the role of genetic mutations in the multistep process of ASAH cannot be tested at present.

In conclusion, our results show that ASAH is a multistep process with four successive steps of risk, with the exact nature of these steps still unknown. These steps may include genetic variations, such as high polygenic risk scores, modifiable clinical factors, epigenetics and somatic mutations. The identification and understanding of these steps may lead to the ability to act on them.

## AUTHOR CONTRIBUTIONS

**Han‐Sol Chang:** Data curation; writing – review and editing. **Katharina A. M. Hackenberg:** Data curation; writing – review and editing. **Nima Etminan:** Data curation; writing – review and editing. **Jan H. Veldink:** Conceptualization; writing – review and editing.

## FUNDING INFORMATION

This project has received funding from the European Research Council (ERC) under the European Union's Horizon 2020 research and innovation programme (PRYSM, grant agreement no. 852173). This study was supported by the Dutch Heart Foundation (Dekker grant 03‐001‐2022‐0157).

## CONFLICT OF INTEREST STATEMENT

The authors declare there is no conflict of interest.

## DISCLOSURES

JHV reports to have sponsored research agreements with Biogen and Astra Zeneca.

## Data Availability

The data that support the findings of this study are available from the corresponding author upon reasonable request.
